# Mental health experiences of mothers in Jos, Nigeria: An interpretative phenomenological analysis

**DOI:** 10.1177/2050312120970714

**Published:** 2021-04-09

**Authors:** Dung Ezekiel Jidong, Nusrat Husain, Christopher Francis, Maisha Murshed, Ayesha Roche, Tarela J Ike, Haruna Karick, Zubairu K Dagona, Juliet Y Pwajok, Pam P Nyam, Shadrack B Mwankon, Anil Gumber

**Affiliations:** 1 Department of Psychology, Nottingham Trent University, Nottingham, UK; 2 The University of Manchester, Manchester, UK; 3 University of Jos, Jos, Nigeria; 4 Teesside University, Middlesbrough, UK; 5 Federal University Oye-Ekiti, Oye, Ekiti, Nigeria; 6 Sheffield Hallam University, Sheffield, UK

**Keywords:** Children, cultural context, care-giving, diet, postpartum, maternal mental health

## Abstract

**Objectives:**

There is an increasing mental health disease burden in mothers with infants and young children, especially in low- and middle-income countries such as Nigeria. Children of distressed mothers suffer early-life exposure from the effects of maternal distress which contributes to the risk of physical and mental health problems in their childhood and beyond. This study explored mental health lived experiences of mothers in Jos, Nigeria.

**Methods:**

Purposive and Snowball sampling techniques were adopted, and a total of 40 mothers participated with 8 to 11 participants in one of the four focus group discussions. Participants were between the ages of 18 and 43 years, self-identified as mothers with each having a child between the ages of 3 and 48 months. Each focus group lasted approximately 60 minutes and was audio-recorded. Interviews were transcribed verbatim and analysed using interpretative phenomenological analysis.

**Results:**

Three overarching themes emerged from the data set such as (1) experience of persisting psychological distress from the time of labour/birth; (2) cultural practices that influence feelings; and (3) anxiety due to limited knowledge about childcare, access to support and healthy food.

**Conclusion:**

Maternal mental health in Nigeria is under-researched and distressed mothers have limited knowledge about evidence-based early child development. The study recommends developing and testing culturally appropriate parenting interventions in Jos, Nigeria. This is likely to be beneficial for the mother and may also improve child health outcomes.

## Introduction

Nigeria has a population of over 206 million,^1^ with a crude birth rate of 39.53 per 1000 people and on average, a Nigerian woman gives birth to approximately 5.3 children during the period of her childbearing years.^2^ However, Nigeria has one of the highest newborn mortality in the world with about 528 deaths per day which contribute to 10% of the global rates.^3,4^ Most of the mortality and morbidity is due to preventable causes, and women with mental health difficulties during the perinatal period is one of the high-risk groups.^5^ For example, maternal distress has shown to have adverse effects on infant and child health outcomes.^6^

Mental health conditions impact significantly on maternal functioning and prevent the mother from being emotionally available to meet the demands of her child.^7,8^ Poor maternal mental health (MMH) could have lifelong consequences on the children. It predicts stunted growth and adverse neurological development in the children of the affected mothers and disturbances in the mother and child bonding and healthy childhood attachment with long-term adverse effects.^9,10^

The risk factors associated with poor MMH vary across cultures and the world regions. In high-income countries (HICs), psychosocial risk factors have been significantly associated with the onset of depression after delivery. However, there are conflicting results for obstetrics risk factors which may be because health centres are more accessible, and child delivery is relatively safe in these countries.^11^ In low- and middle-income countries (LMICs), obstetric complications have been associated with poor MMH, where the clinical practice is often of poor quality and delivery associated with higher rates of maternal and infant morbidity and mortality.^12^ In essence, maternal depression is one of the most common complications affecting 19.8% of women in LMICs.^13^

One of the challenging factors in the prevention and treatment of mental health problems is the lack of empirical data in LMICs.^14^ In 2008, the World Health Organisation (WHO) reported significantly less access to treatment for severe mental health, neurological and substance misuse disorders in LMICs compared to HICs (76%–85% in LMICs versus 35%–50% in HICs).^15^ Although this report is over a decade old, the current circumstances have not changed much. In 2018, WHO’s report showed that there are fewer than 0.1 psychiatrists per 100,000 population in LMICs compared with an average 11.9 psychiatrists in HICs; and similarly, there are 1.4 nurses providing mental health care per 100,000 population in LMICs compared with 23.5 nurses in HICs.^16^ There is an estimated 20%–30% of the Nigerian population suffering from a variety of mental health problems,^17^ with only one out of five potential service-users accessing care.^15,18^ However, it appears that there is little or no data on the current treatment statistics of MMH care in Nigeria.

Understanding MMH as a case-specific concern is essential to develop an effective treatment.^19^ A recent study investigated Somalian women’s experiences of antenatal care in Norway.^20^ The Norwegian study reported that Somalian women are at higher risk of having poor functional health literacy, less likely to comply with the antenatal care available and less likely to trust the information provided to them.^20^ The findings also highlighted participants’ concerns on the failure of care providers to practice with cultural sensitivity. This includes being asked inappropriate healthcare questions regarding marital status, being stigmatised for their religious clothing, feeling stereotyped about the number of children they had and a general lack of understanding about Somali women.^20^ The participants also expressed feelings of loneliness and not being asked about their wellbeing.^20^ The study identified that the issues raised contribute to the lack of trust shown by Somali mothers towards antenatal care services and emphasised the importance of individualised care to improve service-users’ engagement and satisfaction.^20^ The study showed the value of research, which focuses on understanding and interpreting individual experiences with healthcare systems, to support improvement.

In another study, researchers looked at Ethiopia, India and Vietnam and examined the relationship between adversity, cognitive, social capital and mental distress among mothers with children aged between 6 and 18 months postpartum and found that types of adversity experienced varied by country.^13^ The results from India and Vietnam showed that having an additional older woman member in the household was a protective factor for the MMH of participants; however, there was no significant evidence of the same effect for the Ethiopian sample.^13^ This suggests that understanding the context is essential to develop culturally sensitive care models. However, an observational method adopted by Gausman et al.^13^ may not guarantee causation, and the participants were requested to report significant life events that they believed to be important, which suggests that cultural and social norms could influence how they interpret the severity of the events.

An evolutionary theoretical perspective has related three common predictive factors for maternal depression to lack of social support, problems during pregnancy or delivery and history of psychological problems.^21^ In the context of pregnancy and delivery, complications or birthing injury to the mother or infant are considered factors that contribute to the mother’s perception that the child will require additional effort, and this assumption could trigger anxiety and other forms of distress.^21,22^

Evolutionary theorists argued that sociocultural factors play a significant component in the development of maternal depression.^22,23^ In Africa, reports indicate high rates of depression in recent years, which may be as a result of the loss of traditional support systems due to modernisation and sociocultural change.^23^ One study looked at the individual experience of women giving birth in the rural Kipsigis community in Kenya which is known to maintained many aspects of traditional life.^23^ The community did not make over preparations during the perinatal period but recognised the postpartum period as a time of vulnerability.^23^ The mother’s post-birth period was expected to be for rest, while family members provided support in taking care of the running of the household and her other children. Limited access to resources and lack of social support were also strongly correlated with maternal depression.^21^

Although Harkness’s^23^ study used memories and dreams to interpret culture as a mediator for maternal depression in Kipsigis, the results showed memories were more frequently coded positive in the postpartum period, compared to the antenatal period. This indicates that culturally construed forms of social support and accessible resources during the postpartum period can have a significant impact on maternal wellbeing and the mother’s perception of her childbirth experience.^23^ The study suggests that if maternal depression is mediated by culture and given that society provides the necessary levels of social support and a healthy environment, maternal depression may be prevented.^22^ However, the study does not seem to consider the bidirectional possibility between maternal depression and distressing life events, in that maternal depression may also influence factors such as complications during and post-pregnancy.

Beliefs and emotional responses towards motherhood and illness seem to have significant implications on maternal health outcomes. One study identified that participants strived to be good mothers but having maternal depression was associated with the belief that they were not good mothers.^24^ Even though the participants were not sure of the causes and duration of their illness, still, they engaged in coping mechanisms, such as taking psychotropic medication, which indicate positive health-seeking behaviour despite their negative beliefs.^24^ Understanding the thoughts and feelings of those with lived experience provides a valuable insight into personal beliefs about the self as a mother, MMH problems and treatments.

Reviewed literature could be critiqued in numerous perspectives. For example, the support provided to mothers in Kipsigis’s study^23^ does not seem to consider the quality of care as variance, and also, depends on the mother trusting those in her immediate environment. Moreover, in the Norwegian study,^20^ the mothers reported not trusting the healthcare workers, and therefore, perceived the care provided as not satisfactory. Furthermore, much of the research discussed did not look at the onset of poor MMH, and there is lack of research on causation^20^ and there is a possible implication for reverse causation in that the maternal distress may have contributed to the correlated events. This study aims to explore the factors associated with maternal distress to help mothers to better understand how they make sense of their lived experiences in their current health and sociocultural context in Nigeria.

## Method

### Design

An interpretative phenomenological analysis (IPA) was adopted to explore the lived experience of mothers in Jos, Nigeria. Data analysis and interpretations were underpinned by IPA theoretical features such as phenomenology, hermeneutic and idiographic.^25^

### Data analysis

The study employed the IPA method of coding, analysis and interpretation of data to help capture the essential and experiential quality of the account of participants in the data sets.^26^ To identify nuance and participants’ interpretative narratives, inductive and interactive processes were carried out by three researchers who listened and re-listened to the interview audio files, familiarised with the transcripts and made notes of initial exploratory comments; these included linguistic, descriptive and conceptual comments.^26^ Codes were harnessed and processed to identify preliminary themes in each focus group transcript, and the same process was adopted for all transcripts to generate themes. Re-occurring themes were collapsed and refined into the master themes. Master themes were reviewed across entire data sets; the best themes that encapsulate the participants’ experiences were chosen as final themes. Previous studies have consistently shown that IPA provides valuable insight into participants’ perceptions of their subjective lived experiences.^25,26^

### Participants and data collection

Purposive and snowball sampling techniques were adopted for participants’ recruitment in four primary health care (PHC) facilities situated in four local government areas of Plateau State Nigeria. Four face-to-face focus groups were conducted with a total of 40 participants with no dropout or withdrawal of participation. The demographic breakdown of the participants by sites of data collection were Barkin Ladi (n = 11), Jos-South (n = 11), Jos-North (n = 10) and Riyom (n = 8). The four PHC facilities were purposively selected to plan for a future randomised controlled trial. The women were recruited when they brought their newborn for health checks such as immunisation against childhood communicable diseases at the PHC facilities. The sites of data collection were predominantly Berom ethnic people which might have provided data homogeneity based on similar cultural values and traditions. The focus group interviews were conducted between November 2019 and January 2020.

The participants’ inclusion criteria included women who had children (0–48 months); aged 18 years and above; residents of Jos and environs; able to speak and understand English language; and able to provide consent for their participation. Exclusion criteria include women who are non-residents of Jos and environs; less than 18 years; unable to consent; patients currently undergoing treatment for severe mental illness such as psychosis; and unable to speak the English language fluently.

Participants recruited for this study were between the ages of 21 and 43 years (mean age = 32.60 years; standard deviation = 14.79) and each participant’s youngest child was between the ages of 3 and 48 months (mean age = 23.93 months; standard deviation = 6.78). Each focus group interview lasted approximately 60 minutes and were all recorded with a digital audio recording device and transcribed verbatim. Data saturation was reached at the end of the fourth focus group discussion when no more new themes were being generated, and the interviews were stopped.

A pilot focus group was initially conducted with seven volunteers who constituted about 17.5% of total participants. The pilot study helped to examine the face validity and compatibility of interview schedule items with the sample population. The pilot discussions provided a general impression on how the groups felt and thought about the topic items concerning their experiences. The valuable feedback helped to modify questions in the interview schedule. Interview questions were iteratively developed and refined by the research team (see the supplementary material for sample interview questions). The focus group interviews were planned and conducted by a PhD holder and supported by two research assistants (RAs) with MSc and BSc psychology levels of education. The two RAs were both trained in qualitative research methods before the commencement of the focus group interviews.

Interviewers made concerted efforts to explore participants’ deeper meanings with regards to mental health distress such as anxiety and depressive experiences in the postpartum and maternal early childhood periods. However, participants were more keen to discuss the role of family members and sociocultural practices that influenced their overall mental health and wellbeing.

### Ethics

Ethical approvals for the study were received from Nottingham Trent University, UK and the Jos University Teaching Hospital, Nigeria. Participants were initially contacted during their visits on routine clinic appointments in the participating primary health care facilities. Before participation, all participants read the participants’ information sheet and signed the written informed consent forms. All identifiable information in the data sets were deleted or anonymised with pseudonyms.

## Results

**Figure 1.**
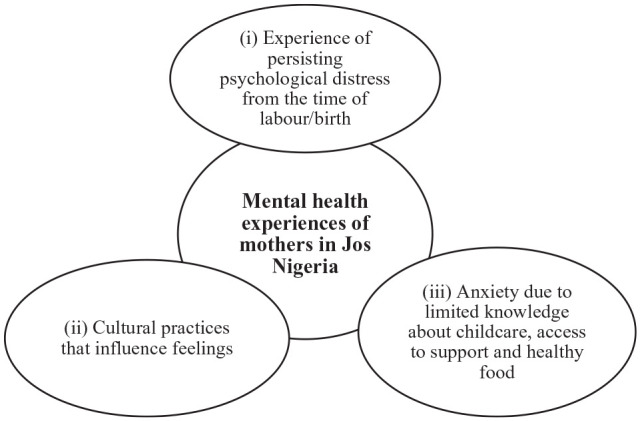
Themes that emerged from the analysed data.

Analysed data sets revealed three overarching themes associated with maternal distress in Jos, Nigeria, as shown in Figure 1.

### Experience of persisting psychological distress from the time of labour/birth

Aside from the excitement of having a newborn baby, data sets showed that mothers’ experiences following childbirth could be quite intense and demanding, both emotionally and physically. Simi (22 years) said:

I can’t sleep even the pain itself, during labour is enough source of stress to me, and you know if you are stress up, so many things will come to your mind, the way you think may change and everything easily makes me angry.

Simi translates her experience of childbirth trauma to having sleep difficulties due to the pain and stress of labour. The continuing pain could be interpreted as a trigger of emotional distress that she expressed in the form of ‘displacement’ and frustration with the happenings around her. At the time of data collection, Simi had a 24-month-old baby, which suggests that maternal experience could be traumatising to the mother for a considerable period of time. However, Kachollom (25 years), who had a 9-month-old baby at the time of data collection, said:

Because I went through a lot of pain going through CS [Caesarean section] and this experience was something else, I find it difficult to sleep, eat, sit due to the pains for months, and combining the work of child upbringing and other domestic chores alone is a big source of stress so it’s not easy.

Kachollom portrayed her emotional distress as a manifestation of the traumatic experience of the Caesarean section and the demands of child upkeep as the mother of a 9-month-old baby. Although, there is an understanding that mothers of a newborn require some rest and reasonable care to enable speedy recovering and healing from childbirth, however, sometimes cultural expectations may require mothers to be brave and should not show any signs of distress. Perhaps, mental health-seeking behaviours may be construed as a sign of the mother’s weakness and lack of bravery.

### Cultural practices that influence feelings

Data analysis revealed traditions, cultural practices and expectations that appeared unique to the period after childbirth. Aside from mutual support from other family relations, there are particular cultural expectations from the grandmother of the newborn. For example, Nvou (27 years), who had a 36-month-old baby at the time of data collection, said:

In our culture, each time I gave birth to my child, my mother-in-law comes to perform the necessary rituals that are culturally bound to keep the state of my health and my baby. She [grandmother of newborn] usually stays with me for a period of 6 months.

The above extract suggests that physical and emotional support from the grandmother of the newborn fulfils tradition and cultural expectations. Nvou had enjoyed the cultural rituals several times she had given birth, especially keeping herself well and her baby. However, 6 months of quality time from grandmothers who may be still in active service or career-driven may be a great luxury that not many women can enjoy. Although Nvou did not specify the exact content of the cultural ritual, she received, however, the following extracts from the same focus group may give more details on the nature of traditions and cultural practices of supporting mothers in their postpartum period. Some participants in the focus group said:

Yes, me too! Because when I gave birth my mother used to bath me with some local herbs boiled with hot water and use it to massage my tummy for many weeks it helped in melting the accumulated blood from the body. I was drinking kunun samiya [local herbal drink] too, helps to clean my body. (Yop, 27 years)

Use of pawpaw leaves and bitter leaves when cooked and allowed to cool. The water is very important in bringing about the overall wellbeing of the baby and her mother. It goes a long way in solving stomach problem. In my culture, we use udah and uziza leaves, it is usually prepared with stockfish, it is believed that it will clean the system especially the accumulated blood in the body. (Kangyang, 33 years)

What is central in the above extracts is the use of cooked herbs for both simple physio- and meal-therapies that are tailored for women in their postpartum period. Both Yop and Kangyang believed that herbal combinations are helpful and beneficial to their health and their babies’ wellbeing. However, the herbal combinations and their effects as mentioned in the above extracts might be used with caution due to little evidence of their efficacy. However, more group members narrated their experience of help they received from the traditional healing system, which they believed to be for a culture-specific ailment. For example, Hwarwat (34 years), who had a 48-month-old child at the time of this focus group discussion, said:

When I gave birth to my [youngest] child because there is a particular ailment I was treated [for], during and after delivery which has defied all medical interventions [all western treatment, drugs and medications] and when I started taking cultural herbs there was a great improvement because after my child delivery there are domestic chores I couldn’t do for several months but now I can do them perfectly.

Although, Hwarwat’s experience of postpartum and maternal ailment was over 4 years at the time of data collection, yet she was confident in narrating her experience of receiving healing with cultural herbs as helpful and compatible after a significant period of trying western medications and treatment that did not help. Following her healing, Hwarwat also self-assessed her wellness using her ability to engage in self-care and also caring for her baby.

In another extract on cultural practices, Weng who had a 12-month-old baby at the time of data collection narrated her experience about some unhelpful postpartum cultural practice of drinking local brew with addictive alcohol content believed to boost breast milk production for the newborn:

like the cultural drink Nzorkokol, I was asked to drink it too with a high concentration of salt, believing it will boost breast milk production, although it worked as there was enough breast milk for my baby, but the side-effects I experienced was highly terrible as all my eyes and face swelled up for over two days later. (Weng, 32 years)

As shown in the above extracts, Weng believed that as a nursing mother, she could produce enough breast milk for her baby with a simple consumption of local brew and/or salt, which is fascinating. However, some of these practices are built on superstitious beliefs due to limited evidence in its usage and effectiveness, and this may have severe health complications. Addiction and excessive salt consumption could have adverse effects on the health and wellbeing of nursing mothers and their children.

### Anxiety due to limited knowledge about childcare, access to support and healthy food

Knowledge and skills of care of the newborn and good nutrition for both mother and child are essential in healthy child upbringing, especially for first-time mothers with no prior experience. For example, Zere (22 years), who had a 3-month-old baby at the time of data collection, narrated her experience as a first-time mother, said:

My major difficult experience is on how to bath the child, and there was no support from my mother, mother-in-law or any relatives. I felt bad and I believe that experience is not the best for one to go through.

Zere lamented on her lack of knowledge of how to perform basic tasks such as bathing her baby and calling it a terrible experience that no other mothers should go through such difficulties. The extract also suggests that the mother’s inability to provide routine care for their babies could be a potential risk factor for maternal distress. This may also imply that if there were maternal training on essential childcare for mothers, such as Zere, it might be beneficial as a first-time mother on how to be self-reliant. Furthermore, the results show that mothers were concerned about healthy food, good sleep and personal hygiene, and these were reported across the focus groups. For instance, Veirat (23 years) narrated her experience of care she provides to her 9-month-old baby in the following extract:

I will like to maintain personal hygiene like keeping myself and my baby clean, eating a balanced diet including fruits and vegetables is very good in healing and rebuilding the body [. . .] even sleeping at the appropriate time but it is difficult with the small-pikin [little child].

Veirat expressed her experience in the above extract as having an awareness of good hygiene for self and baby care, healthy eating habits, good sleeping cycle and the potential impact on her physical and mental health as well as the wellbeing of her baby. Moreover, Mwere (27 years), who is a mother of 36-month-old baby, suggested that ‘a well-fed mother could translate into healthy baby’:

Yes! If the baby is strong and growing well it means that the mother is feeding well too but, some of us mothers depends on our farms and cultural food like Kunu [a local indigenous drink made of grain] that we drink day in, day out, it affects the baby’s growth. Although, my gwote and acha (indigenous vegetable soup) helps me a lot in the growth of my child.

Although Mwere acknowledged the position of Veirat on the notion that a healthy mother will equate to a healthy child but lamented on her continuous dependence on indigenous local drink also known as ‘kunu’ which in itself alone may not be a balanced diet of healthy food, yet, aware of the mixtures of other local dishes may be beneficial, however, Mwere is worried that deprivation and limited access to healthy foods and balanced diets may cause child malnutrition. It is apparent that continues worrying and anxiety about lack of access to essential resources for postpartum and maternal early childhood survival could be a potential source of depression and other maternal distresses.

## Discussion

This study explored postpartum and maternal lived experiences of mothers in Jos, Nigeria. Findings revealed three themes in relations to the study’s research questions. The themes include (1) experience of persisting psychological distress from the time of labour/birth; (2) cultural practices that influence feelings; and (3) anxiety due to limited knowledge about childcare, access to support and healthy food. A common finding across the four focus groups was a persisting and enduring psychological distress since birth. The experience of difficult childbirth labour appeared to be re-occurring with flashbacks triggering emotional distress. Similarly, Molloy et al.^8^ recently explored the perceptions of parenting after birth trauma. They reported that mothers who suffered birth trauma are at higher risk of anxiety and fear of their child’s health and their parenting capability.

The current findings are consistent with the previous studies,^27,28^ which reported long-term consequences of postpartum distress and its adverse impact on child development, disturbances in the mother and child bonding and poor attachment. This finding is supported by both Hagen^21^ and Tracy’s^22^ study. Mothers who are distressed are less likely to be sensitive to child’s cues and exhibits less emotional availability, which is associated with poor bonding.^21^ In this study, mothers are portrayed as someone who celebrates the birth of their newborn but later feels overwhelmed by postpartum distress that affects their ability to cater for their newborn.

Our study shows that some cultural practices may enhance positive emotional feelings for mothers of newborn children. In specific, there are cultural expectations of the grandmothers to provide specific physical and emotional support to their daughters and the newborn for about 6 months. The positive role of grandmothers in this study was partly explained in the study by Gausman et al.^13^ which reported a lower risk of postpartum depression in the Indian and Vietnamese’s households with older persons. However, this was not the same as the Ethiopian mothers, which is contrary to the present findings. This study showed that cultural practices and traditions are essential in alleviating maternal distress. Similarly, Harkness^23^ showed that support systems are culturally construed for all forms of social support and accessible resources during the postpartum period, which has a significant impact on the wellbeing of mothers and their perception of the childbirth experience.

In specific, the current findings showed efforts by mothers to seek the help of some sort and the use of cultural remedies such as herbs and indigenous foods in coping with postpartum distress. This is similar to the study conducted by Patel et al.,^24^ which suggested that, despite negative thoughts of postpartum challenges, mothers were utilising coping strategies and health-seeking behaviours. This study and that of Patel et al.^24^ and many others show that mothers strive to be good parents. Despite the potentially favourable impact of cultural practices and traditions, there is limited empirical evidence of such cultural practices, therefore, caution is required as some of the cultural practices are carried out based on superstitious beliefs. These could be unhelpful and even dangerous to the health and wellbeing of both the mother and the child.

Our results show that some mothers, after childbirth, may not be well-equipped with the basic knowledge of childcare. Despite the promising cultural practice of experienced family members offering care for a considerable period, yet, every mother needs to have the necessary independent skills of childcare as such support may not always be available. This finding is similar to the outcome of a recent study by Utne et al.,^20^ they reported that Somalian women in Norway are more likely to have poor functional health literacy which may have adverse effects both on the health and wellbeing of mother and child. Although, this may not be a direct reflection of this study’s context as the Utne et al’.s^20^ study was conducted with Somalian women in Norway which is a different country from their cultural heritage. Our findings suggest a need for more parenting skills and education around both self and child care. The results of this study can provide some insight into the postpartum and maternal lived experiences, which can help develop culturally sensitive interventions.

This study has two fundamental limitations. First, the sites of data collection are predominantly of Berom ethnic women and culture, which does not reflect other ethnic women in Jos Plateau State, where data were collected. However, this limitation has helped the study to focus on harnessing data from communities that shared similar cultural values and traditions. Second, the study explored MMH lived experiences; however, participants were not mental health service-users, and we did not examine psychological distress using any validated measures which is a limitation. This limitation could be addressed in future studies to examine maternal lived experiences of mothers receiving mental health care in these communities. This study’s strength lies in its exploratory nature, and the use of IPA theory and philosophy to harness rich and in-depth knowledge of mothers’ postpartum and maternal lived experiences. This is essential in developing new knowledge that can serve as the basis for developing a new model of intervention.

In conclusion, this study aimed to generate and set the basis for new knowledge about maternal lived experiences, during and after childbirth, and to further give voice to mothers who have previously been unable to share their lived experiences. Using focus group interviews, mothers spoke freely about their lived experiences, and the researchers elicited rich and in-depth information. More practically, a qualitative approach, therefore, captures the diversity, idiosyncrasy and complexity of motherhood in Jos, Nigeria. This study showed that distress of difficult childbirth labour could persist for a long time. However, help from family members was expressed as an essential source of physical and emotional support. Although traditional and cultural practices shaped mothers’ experiences, however, some of the practices were potentially based on superstitious beliefs and would require further research. Finally, in terms of practice, policy and research, this article provides local service-providers with valuable evidence on ‘taken-for-granted’ experiences such as emotional distress due to limited knowledge of childcare and anxiety associated with limited access to healthy food for the mother and child. This research sets a foundation for future studies and calls for intervention research to empower mothers with culturally appropriate skills that are beneficial for childrearing and alleviating MMH difficulties such as anxiety and depression.

## Supplemental Material

sj-doc-1-smo-10.1177_2050312120970714 – Supplemental material for Mental health experiences of mothers in Jos, Nigeria: An interpretative phenomenological analysis Click here for additional data file. Supplemental material, sj-doc-1-smo-10.1177_2050312120970714 for Mental health experiences of mothers in Jos, Nigeria: An interpretative phenomenological analysis by Dung Ezekiel Jidong, Nusrat Husain, Christopher Francis, Maisha Murshed, Ayesha Roche, Tarela J Ike, Haruna Karick, Zubairu K Dagona, Juliet Y Pwajok, Pam P Nyam, Shadrack B Mwankon and Anil Gumber in SAGE Open Medicine

sj-pdf-2-smo-10.1177_2050312120970714 – Supplemental material for Mental health experiences of mothers in Jos, Nigeria: An interpretative phenomenological analysis Click here for additional data file. Supplemental material, sj-pdf-2-smo-10.1177_2050312120970714 for Mental health experiences of mothers in Jos, Nigeria: An interpretative phenomenological analysis by Dung Ezekiel Jidong, Nusrat Husain, Christopher Francis, Maisha Murshed, Ayesha Roche, Tarela J Ike, Haruna Karick, Zubairu K Dagona, Juliet Y Pwajok, Pam P Nyam, Shadrack B Mwankon and Anil Gumber in SAGE Open Medicine
