# Supplementary material for: Mental health experiences of mothers in Jos, Nigeria: An interpretative phenomenological analysis
Source: SAGE Open Med. 2021 Apr 9;9:2050312120970714. doi: 10.1177/2050312120970714 (PMC8040383; doi:10.1177/2050312120970714)
Supplement: sj-pdf-2-smo-10.1177_2050312120970714 – Supplemental material for Mental health experiences of mothers in Jos, Nigeria: An interpretative phenomenological analysis [file sj-pdf-2-smo-10.1177_2050312120970714.pdf]

Sample interview questions

(i) Would anyone like to kindly share their experiences of wellbeing following childbirth?
Prompts: (a) What are some of your best experiences after childbirth?
(b) Were there any feelings of emotional distress? If yes, what are the feelings and what do you think are some of the reasons for these feelings?
(c) What do you think may help in promoting better mental health and wellbeing during the periods after childbirth?

(ii) Do you think your mental health experiences after childbirth could be associated with your cultural practices?
Prompt: Are there typical cultural values and practices you think were essential in your mental health experiences after childbirth?

(iii) Do you think your postpartum mental health experiences affect your child's growth and development in some ways?
Prompt: If yes, in what ways does it affect your child?

(iv) Where do you think other mothers would prefer to get help or support for mental health and wellbeing?

(v) Can you share your experiences of accessing mental health and wellbeing support?

Prompt: What would you say are your major challenges of accessing mental health and wellbeing support?

(vi) Is there anything else you would like to share about your mental health experiences after childbirth that was covered in the group discussion?
